# Dynamic Quantitative Intravital Imaging of Glioblastoma Progression Reveals a Lack of Correlation between Tumor Growth and Blood Vessel Density

**DOI:** 10.1371/journal.pone.0072655

**Published:** 2013-09-12

**Authors:** Clément Ricard, Fabio Stanchi, Thieric Rodriguez, Marie-Claude Amoureux, Geneviève Rougon, Franck Debarbieux

**Affiliations:** 1 Developmental Biology Institute of Marseille-Luminy (IBDML), Aix Marseille University-CNRS 7288, Marseille, France; 2 European Center for Medical Imaging (CERIMED), Marseille, France; 3 VIB3 Vesalius Onderzoekscentrum, KU, Leuven, Belgium; University of Navarra, Spain

## Abstract

The spatiotemporal and longitudinal monitoring of cellular processes occurring in tumors is critical for oncological research. We focused on glioblastoma multiforme (GBM), an untreatable highly vascularized brain tumor whose progression is thought to critically depend on the oxygen and metabolites supplied by blood vessels. We optimized protocols for orthotopic GBM grafting in mice that were able to recapitulate the biophysical constraints normally governing tumor progression and were suitable for intravital multiphoton microscopy. We repeatedly imaged tumor cells and blood vessels during GBM development. We established methods for quantitative correlative analyses of dynamic imaging data over wide fields in order to cover the entire tumor. We searched whether correlations existed between blood vessel density, tumor cell density and proliferation in control tumors. Extensive vascular remodeling and the formation of new vessels accompanied U87 tumor cell growth, but no strong correlation was found between local cell density and the extent of local blood vessel density irrespective of the tumor area or time points. The technique moreover proves useful for comparative analysis of mice subjected either to Bevacizumab anti-angiogenic treatment that targets VEGF or to AMD3100, an antagonist of CXCR4 receptor. Bevacizumab treatment massively reduced tumoral vessel densities but only transiently reduced U87 tumor growth rate. Again, there was no correlation between local blood vessel density and local cell density. Moreover, Bev applied only prior to tumor implantation inhibited tumor growth to the same extent as post-grafting treatment. AMD3100 achieved a potent inhibition of tumor growth without significant reduction in blood vessel density. These results indicate that in the brain, in this model, tumor growth can be sustained without an increase in blood vessel density and suggest that GBM growth is rather governed by stromal properties.

## Introduction

The development of dedicated imaging modalities and appropriate animal models is needed to characterize cellular interactions *in vivo* [1] and to evaluate drug effects on specific cell populations [2]. We chose to focus on glioblastoma multiforme (GBM) an invariably fatal brain tumor accounting for approximately 40% of all primary malignant brain tumors. GBM are highly vascularized tumors, and preclinical data have suggested that GBM growth critically depends on the generation of tumor-associated blood vessels [3], [4]. In particular, the vascular endothelial growth factor (VEGF) pathway has been a prime drug target underscored by the approval of bevacizumab (Bev, an anti-VEGF monoclonal antibody) for treatment of patients [5], [6], [7]. The effect of anti-VEGF on tumor cells that secrete high levels of VEGF [8] is however transient [9], [10], [11] and the majority of patients eventually relapse. The mode of action for its clinical benefits is still not fully understood and needs further investigations on preclinical models. The size of tumor burden, its molecular heterogeneity as well as the stage of development can be responsible for variability in disease outcome. Therefore, a better understanding of tumor physiology first requires studying analytically the influence of several parameters on simple models before studying more realistic ones.

Whereas MRI is currently used to follow GBM progression, the heterogeneity of patient populations, the sparseness of exams as well as the approximate relationship between tumor contrasts and actual tumor cell density ultimately lead to misinterpretations [12], [13], [14]. Consequently, there is considerable need to develop dedicated imaging modalities and appropriate animal models to investigate *in vivo*, the drug effects on specific cell populations with the aim to optimize the efficacy of treatments in humans. To evaluate interactions between tumor cells and tumor vessels at single cell resolution and over time *in vivo* we used intravital two-photon microscopy in an orthotopic xeno or allotransplanted mouse models. Traditionally, *in vivo* two-photon imaging of the murine central nervous system has either involved the use of open-skull or thinned-skull preparations [2], [15]. While the open-skull technique is versatile, it is intrinsically suboptimal for studying GBM because it is invasive and leads to tumor growth outside of the dura mater when grafted at imaging relevant depth. The thinned-skull approach is minimally invasive, but the repeated re-thinning of skull required for chronic imaging increases the risks of tissue injury and allows for a limited number of imaging sessions. A polished and reinforced thinned skull technique was recently implemented [16] but light diffusion and absorption at the interface with bone compromise spatial resolution and limit imaging depth.

We presented here a chronic window method for monitoring GBM growth and angiogenesis in the first millimeter below the dura and over an extended period of time. Importantly, whereas GBMs are heterogeneous with hypervascularized inner regions and peripheral regions from which the tumor cells invade the normal brain, our technique analyze most of the tumor volume at once while preserving a fully intracerebral growth. We established the methods for an appropriate quantification of such images that were repeatedly acquired from the same subjects to collect dynamic data. The weak correlation observed between tumor progression and blood vessel density was further evaluated under systemic pharmacological manipulations that further uncoupled the modulation of tumor cell densities from that of vascular density. Using two FDA approved molecules Bev and AMD3100 [5], [17], [18] which both inhibited tumor growth despite differential actions on vascularization, we provide evidence that GBM can develop without angiogenesis. A possibility is that both anti-tumor drugs mainly act on the stroma to impair tumor progression.

## Materials and Methods

### Animal care guidelines

In accordance with the policy of the Developmental Biology Institute of Marseille-Luminy (IBDML) and the French legislation, experiments were done in compliance with the European Community Council Directive of November 24, 1986 (86/609/EEC). The research on animals was approved by the Direction Départementale des Services Vétérinaires des Bouches-du-Rhône (permit number 13.055.21) and has been approved and authorized by the National Committee for Ethic in Animal Experimentation (Section N°14; project 87-04122012). Six to seven weeks old (male) NIH nude mice (Foxn1-null, n = 75, Charles River, France) and C57 Blak6 (n = 6) were housed in cages with food and water *ad libitum* in a 12 h light/dark cycle at 22±1°C.

### Cell culture and gene transfection

U87 MG cells (ATCC number HTB-14, P133) were transfected with a plasmid for GFP expression (pEGFP-C1, Clontech, Mountain View, USA). Cells that stably express GFP were selected using Geneticin (0.5 mg/ml, Gibco). Five passages following transfection, cells were massively expanded in the presence of Geneticin and aliquoted prior freezing. U87MG-GFP cells were cultured as monolayers in MEM+Earle's+GlutaMAX-1 (Gibco) supplemented with 10% Fetal Calf Serum (Thermo Scientific), 1 mM pyruvate (Gibco) and 0.1 mM non-essential amino-acids (Gibco). Cells were kept at 37°C in a 5% CO2 atmosphere.

For each animal series, an aliquot was unfrozen and plated to reach confluence two days before their implantation in mice. Confluent cells were then replated on Petri dishes coated with 0.75% soft agarose to induce aggregation of the cells into spheroids due to physical interactions with the substrate. Conditioned medium was added to the fresh culture medium (1/4th volume). Spheroid with diameters of 150–200 µm were identified and sucked into the tip of a glass pipette with a few µl of culture medium. Pipette inner diameter was used to size spheroids.

GL261 cells, a murine glioma cell line (National Cancer Institute, Charles River Labs), were transfected with a plasmid for DsRed expression (pDsRed2-N1, Clontech, Mountain View, USA). Clones that stably express DsRed were selected using Geneticin (0.5 mg/ml, Gibco). Cells derived from one clone were cultured as monolayers in RPMI1640+GlutaMAX-1 (Gibco 61870) supplemented with 10% Fetal Calf Serum (Thermo Scientific) and Geneticin (0.5 mg/ml, Gibco). Five passages following transfection, cells were massively expanded in the presence of Geneticin and aliquoted prior freezing. Cells were unfrozen when needed and kept at 37°C in a 5% CO2 atmosphere.

### Animal model

**Figure 1:**
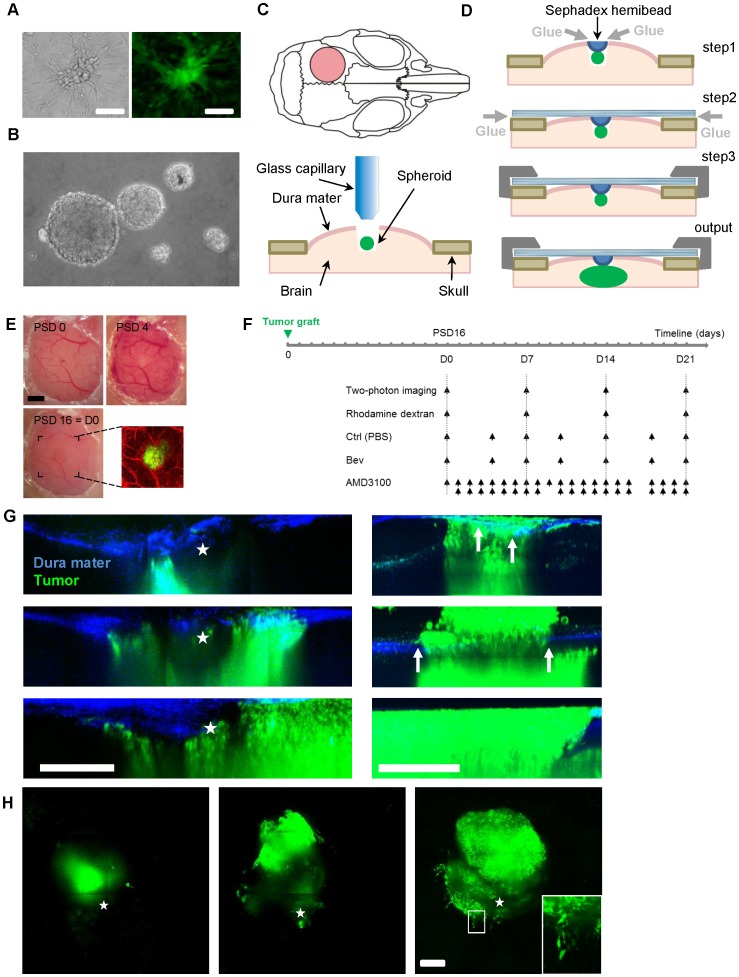
A truly intracerebral GBM model suitable for two photon imaging of tumor cell and blood vessels. U87-GFP cell cultures, on plastic (**A**) and forming spheroids on agarose substrate (**B**). (**C**) Drawings showing the position of the cranial window on the mouse parietal bone (pink circle) and tumor grafting: after craniotomy, spheroids of 150–200 µm diameter where injected focally ∼250 µm below brain surface without removal of the dura mater (**D**) Dura sealing and glass window fixation: a Sephadex hemi-bead (blue) is glued (gray arrows) to the dura to clog the pipette trajectory (step1); the glass window is glued (gray arrow) to the surrounding bone in close contact with the dura (step2); the cement (dark gray) is used for long term seal to the skull (step3); the tumor is forced to develop inside the physically constrained brain parenchyma (output). (**E**) Progression of the acute inflammatory vascular response from the installation of the cranial window on post-surgery day 0 (PSD0) to PSD16. PSD16 corresponds to the day of first visualization (D0). A bicolor two-photon projection image is presented on the right. (**F**) Scheme summarizing the time lines of application of Bev, AMD3100 and vehicle with regard to the date of 2P observations. (**G**) Sagittal maximal intensity projection over the tumor (green) showing the spheroid 6 days post implantation and its evolution at 2 weeks intervals. Second harmonic signal from the dura mater is blue. Position of the Sephadex bead is outlined (star). The tumor cells remain below the dura mater into the brain parenchyma (left). When dura mater is not sealed extradural growth of the tumor is already detectable 6 days post implantation (arrows) and further developed over time. Extradural tumor progression is 3 times larger than that observed inside the brain and after 4 weeks the proliferation that occurs at the interface between the glass and the dura mater prevents further imaging of the tumor growing inside the brain (right). (**H**) Horizontal maximal intensity projection over the whole tumor volume of the same image stacks as for **G** (left). Single cell resolution is achieved (Inset: zoom of the white ROI). Scale bars: all scale bars: 300 µm, E: 1 mm & H inset 25 µm.

Mice were anaesthetized by intraperitoneal injection of a mixture of Xylazine/Ketamine (12 mg/kg and 120 mg/kg, respectively) and positioned on a stereotactic frame (Mouse adapter, Harvard Apparatus). A square craniotomy (diameter: 3–4 mm) was performed on the left parietal bone. An injection hole was performed in the dura using a 31G needle to prepare insertion of the glass pipette. The spheroid of U87 cells was then pressure injected in the cerebral cortex approximately 250 µm below the brain surface using micromanipulators. Following implantation, a moist half-Sephadex bead with diameter fitting with the dura opening was inserted in the injection wound and glued to the dura-mater and surrounding parenchyma using histo-compatible acrylic glue (Cyanolit). A round glass coverslip (diameter: 6 mm) was then glued on the surrounding bone that had been previously thinned to ensure close contact between glass and brain. Glass window was further fixed to the skull by dental cement (**Figure 1C–D**). Anti-inflammatory treatment was administered by means of subcutaneous injections of Dexamethasone (0.2 mg/kg, Vetoquinol) and Carprofen (5 mg/kg, Rimadyl Pfizer) every 48 h for two weeks to help resuming post-surgical inflammation. Animals were first imaged when inflammation was resolved on day 16 post surgery as indicated by normal vascular pattern (**Figure 1E–F**).

### Microscopy

Mice were anaesthetized by an intraperitoneal injection of a mixture of Xylazine/Ketamine (10 mg/kg and 100 mg/kg, respectively) and injected intravenously with 100 µl of a RhodamineB conjugate dextran (70 kDa) solution (20 mg/ml in PBS, R9379, Sigma Aldrich) prior to each imaging session and positioned on a stereotactic frame. Height of each earbar and of the mouth piece was adjusted independently under microscope control to ensure planar repositioning of the animal brain surface based on visual landmarks. Anesthesia was supplemented hourly (40 mg/kg; 4 mg/kg) to prevent toe pinch reflex.

**Figure 2:**
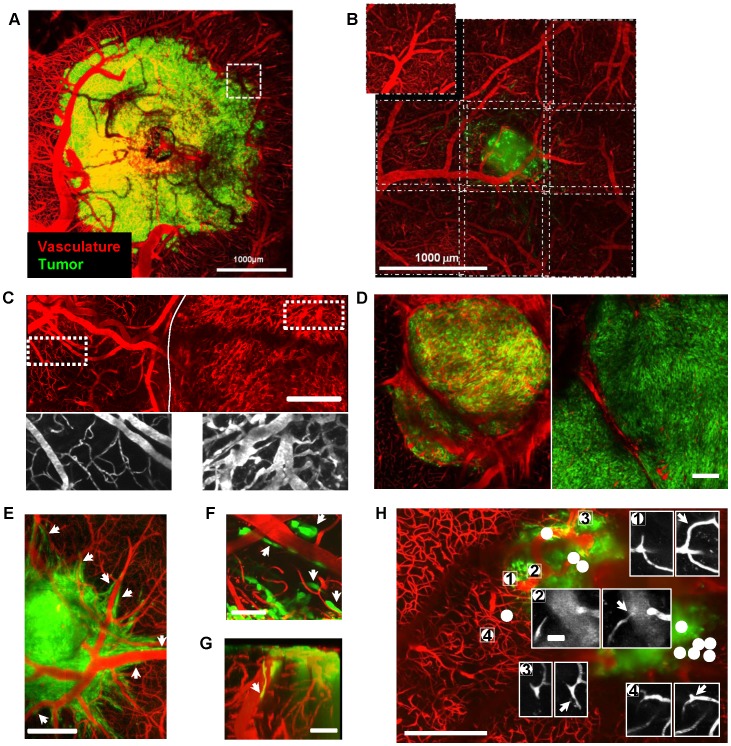
Imaging blood vessel network and interactions with tumor cells. (**A**) Mean projection image of a tumor and associated vascularization acquired through a 20× Water Immersion objective (N.A. = 1.0). One field of view corresponds to the dotted line box; 49 such fields were acquired to cover the full tumor extent. (**B**) For quantitative pharmacological experiments, a 10× dry objective (N.A. = 0.45) was used to increase data throughput by a factor 4. An overlap of 50 µm between fields of view ensured qualitative image stitching performed with ImageJ 3D Stitching macro, “pcm3D”. (**C**) Upper part, 3D projection of the vascular network over a depth of 150 µm of the tumor (right of the plain line) and of surrounding healthy brain (left of the plain line). Lower part: zooms of the outlined ROIs. Thin and straight normal brain vessels (left); tortuous and swollen tumor vessels (right). (**D**) Example of bicolor images showing at a 10 day interval the average vascular density (red) and tumor cell density (green) in a 25 µm thick horizontal section of the tumor located 200 µm below the brain surface. Note that the lower vascular density observed after 10 days is not associated with a reduction of tumor cell density. (**E,F**) Horizontal mean projection images over 100 µm subdural layers 15–19 days post implantation showing perivascular invasion (arrow heads). (**G**) Sagittal 3D rendering projection showing superficial tumor cells (arrow heads) migrating toward deeper brain regions along a large vertical vessel. (**H**) Mean projection of the volume of interest scanned for vascular changes occurring at a 1 day interval. All the changes (white dots) were found inside or within 150 µm off the tumor margin but none in the healthy brain region. Insets show examples of microvascular changes (arrows) at several locations. Scale bars, A&B: 1 mm; C&D: 300 µm, F&G: 50 µm,; H:300 µm, inset 25 µm.

We used Zeiss two-photon microscopes (Axioskop 2FS LSM510 or LSM7MP) home-modified to allow animal positioning below the objective and coupled to a femtosecond pulsed infrared laser (Mai-Tai, Spectra Physics) tuned at 840 nm. 10× air objective (NA = 0.45) or 20× water immersion objective (NA = 1.0) were selected based on field of view/resolution considerations (**Figure 2A–B**). GFP (500–550 nm) and RhodamineB (575–640 nm) signals were simultaneously collected on 2 non-descanned detectors. Images were typically acquired over a depth of 500 µm using 5 µm steps. Laser intensity was linearly increased with depth and the same profile of settings was used at all depths for all mice involved in quantitative fluorescence experiments.

### Treatment

A 10 mg/kg dose of Bevacizumab (Anti-VEGF, Roche) in PBS was injected i.v. every 2–3 days from the first day of visualization until the last. A 1.25 mg/kg dose of AMD3100 (CXCR4 antagonist, Sigma Aldrich) in PBS was subcutaneously injected twice daily from the first day of visualization until the last. PBS vehicle injected i.v. was used as a control. Animals groups for treatment were homogenized before starting the pharmacological protocol based on the size of the tumor on the first day of visualization. See **Figure 1F** for time table details.

BrdU 50 mg/kg was administered in 200 µl PBS i.v. 7 days after initiation of Bev treatment and 2 days prior sacrifice.

### Immunostaining

Brain tissues fixed with paraformaldehyde 4% were cryosliced in 25 µm thick sections. Blood vessels were revealed using rabbit anti-Laminin antibody (Novus Biological, 1/1000) and proliferating cells revealed with rat monoclonal anti-BrdU antibody (Abcam, 1/100). Donkey anti-rabbit and donkey anti-rat secondary antibody flagged with Cy3 and Cy5 were respectively used at (1/100) and (1/200). Tiled images of the whole tumor sections were acquired on a Zeiss Axioimager fluorescence microscope with 10× (NA = 0.30) dry objective.

### *In vitro* experiments

U87-GFP cells were seeded in a 96 wells culture plate (7000 cells per well) in (MEM+Earle's+GlutaMAX-1, 10% Fetal Calf Serum, 1 mM pyruvate and 0.1 mm non essential amino-acids).

Cells were divided in 5 sub-groups (n = 6 to 12 wells per sub-group) (day 0) and incubated with the different drugs in the same medium volume: VEGF (50 ng/ml), Bev (250 ng/ml), VEGF+Bev (50 ng/ml and 250 ng/ml, respectively), AMD3100 (10 µg/ml). The culture plate was positioned on an inverted Zeiss Observer Z1 with Colibri fluorescence system equipped with a motorized stage and an incubation chamber (37°C, 5% CO2). Visible and fluorescence images were acquired for each well every 30 min with a 10× (NA = 0.30) objective for 59 to 114 h using Zeiss Axiovision 4.8 software. Images were then processed using the ImageJ free software and the MTrackJ plugin.

### Data analysis

**Figure 3:**
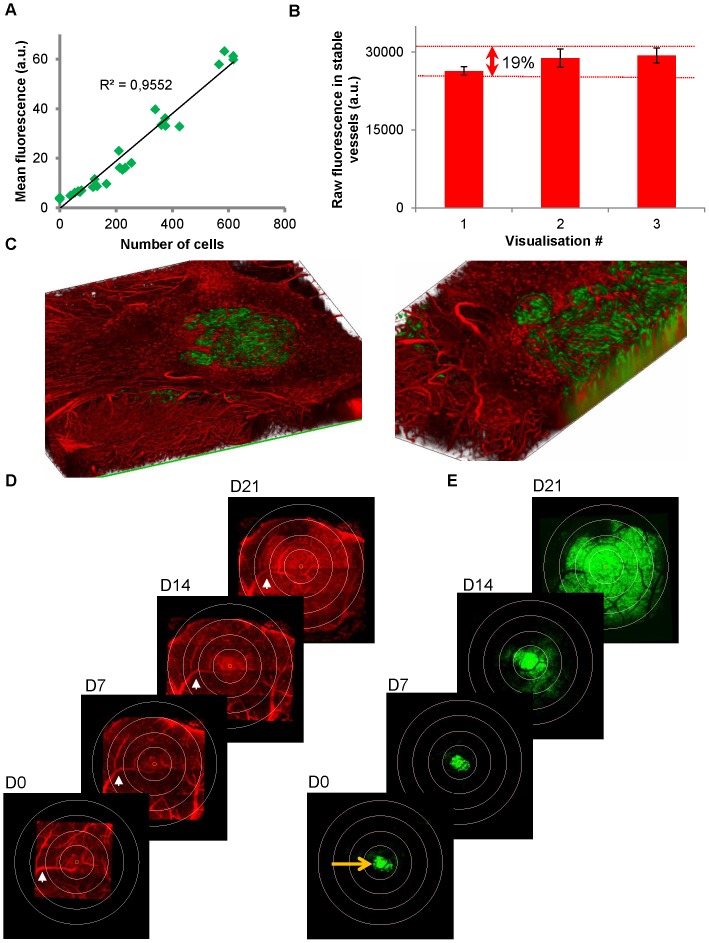
Quantitative fluorescence measurements. (**A**) Average green fluorescence recorded from U87-GFP in culture wells was highly correlated (R^2^>0.95) with the number of cells. Green fluorescence was then used as an index of tumor cell density. (**B**) Raw fluorescence counts recorded after i.v. injection of Rhodamine Dextran in healthy brain of n = 19 to 25 mice over repeated imaging sessions and corresponding SEM. In healthy brain, vascularization is stable and average fluorescence in the raw images varied within 19% over 3 successive visualizations. These variations include all the methodological biases such as inter-animal variability, variations of Rhodamine dextran injected volumes, tilt angle during mouse repositioning, changes of tissue optical properties, laser and detector variability. Stability over time allows for quantitative analysis of our images. (**C–E**) Analysis of 3D images stacks. (**C**) 3D rendering image (left) and zoom (right) of a bicolor 3D stack acquired over a depth of 400 µm and a field of view covering the whole extend of the tumor in the horizontal plane (2.4 mm2). Volumes of interest acquired every week are semi-automatically registered based on several vascular landmarks (example: white arrow) and size adjusted by adding NaN (Not a Number) voxels. The set of 40 images corresponding to the superficial tumor volume between ∼50 µm and ∼250 µm below the brain surface are projected by summation. Then channels are separated to obtain a set of vascular projections (**D**) and its corresponding set of brain tumor projections. (**E**) Coordinates of the fluorescence center of mass in the green projection are then used to locate tumor epicenter on D0 (orange arrow, E). Tumor epicenter is then repositioned on every projection (orange circle in D & E). Concentric circles with 330 µm radius increments are then virtually superimposed on the projections. The area between two successive circles defines a conserved region of interest (ROI) across time points. Evolution of mean fluorescence has thus been followed as an index of mean blood vessel density and an index of tumor cell density in the 4 ROIs.

In vivo image stacks corresponding to individual yet overlapping field of views were stitched together (see **Figure 2A–B**) and semi-automatically registered based on stable vascular landmarks using the sets of plugins named “Align Stacks” and “Stitching” for ImageJ software. A conserved volume of interest ranging from ∼50 to ∼250 µm below the brain surface was extracted for all time points. Green and red channels were then separated and analyzed separately using home-written macros. Each channel was sum projected over the whole volume and tumor epicenter automatically located from the 2D projection corresponding to the first day of visualization (D0). Concentric regions of interest (ROIs) conserved across all time points were defined on the projections for each mouse. Evolution of the mean fluorescence in each ROI was used as an index of local tumor cells density (green) and of local blood vessel density (red) (see **Figure 3** for details). For reliable comparisons, green fluorescence values recorded for each session were normalized for each animal to the one measured at the tumor core (ROI1) on the first day. Similarly, the red fluorescence inside the tumor was normalized to the mean fluorescence in surrounding healthy brain (ROI4).

Images of immunostained sections were manually thresholded to get rid of the background and the densities of thresholded voxels were used as an index of vessel and proliferating cell densities, respectively.

### Statistics

All data are expressed as mean ± SEM. Variation coefficient (VC) was used to estimate the stability and reproducibility of the measurements. Mann-Whitney one tail U-test was used to test for the reduction of cell density and blood vessel density between the treated and control groups. p<0.05 was used as a criterion for significance. The plot of red versus green fluorescence measurements was systematically fitted with a linear regression intercepting the vertical axis at y = 1. Correlation between the data was acknowledged by a p value<0.05 as calculated for a Pearson correlation. The quality of the correlation between cell density and blood vessel density was evaluated and interpreted using classical standards: strong correlation when determination coefficient (R^2^) of the regression was superior to 0.5; poor correlation for R^2^<0.25 (**Figure S1B**). Other types of standard regressions were tested but resulted in no better R^2^ (**Figure S2G**). All statistical analyses were performed with Excel (Microsoft, USA) and GraphPad Prism4 (GraphPad software, USA).

## Results

### A physiological orthotopic tumor model adapted to multiphoton live imaging

We designed a GBM model compatible with two-photon *in vivo* imaging and mimicking physiological tumor development [19], [20]. We prepared homogeneous groups of immunodeficient mice grafted 250 µm deep into the left parietal cortex with GFP-U87 (**Figure 1A–B**) or DsRed-GL261 (**Figure S1**) spheroids. Dura mater was essentially intact everywhere but at the site of injection where it was sealed at the end of the tumor implantation process. Sealing consisted in gluing a Sephadex hemibead to the meninges and surrounding tissue in order to block the pipette trajectory (**Figure 1C–D**). The repulsive Sephadex surface thus prevented tumor cells escape from deep brain layers toward the glass surface, a process that is otherwise responsible for unphysiological tumor cell proliferation in the absence of biophysical constraints (**Figure 1G**). Over time a tumor mass formed and expended in the 3 directions under the intact dura matter with cells infiltrating the peripheral regions as a common histological hallmark of malignant gliomas (**Figure 1G–H**). Imaging protocol started two weeks after grafting (**Figure 1F**) once animals had recovered from the transient surgical vascular inflammation (**Figure 1E**).

### Imaging tumor growth, angiogenesis dynamics and heterogeneity

Rhodamine B dextran (70 kDa) was injected intravenously prior to every imaging session to visualize vasculature (**Figure 2**). Several fields of view (up to n = 49) were acquired to cover the full tumor extent (**Figure 2A** **, Video S1**). An overlap of 50 µm between fields of view ensured qualitative image stitching performed with ImageJ 3D Stitching macro, “pcm3D” (**Figure 2B**). Tumor growth was accompanied by extensive vascular remodeling and the formation of new vessels (**Figure 2C–H**) that were dense, tortuous and swollen compared to healthy brain vessels (**Figure 2C**) as described for GBM patient tissue specimens [17]. Increases of cell density observed over time were not necessarily associated with increases in vascular density (**Figure 2D**) despite tumor cells migrating in close vicinity of blood vessels. Interactions between tumor cells and local blood vessels were sometimes obvious (**Figure 2E–G** **, Video S2**). The ability to follow individual blood vessels by high resolution imaging of the same field of view at 24 h intervals revealed several spots of marked and rapid vascular changes (**Figure 2H**). These plastic spots were exclusively located inside the tumor or within a ∼150 µm margin (**Figure 2H**).

### Quantitative analysis of proliferation and local vessel density

**Figure 4:**
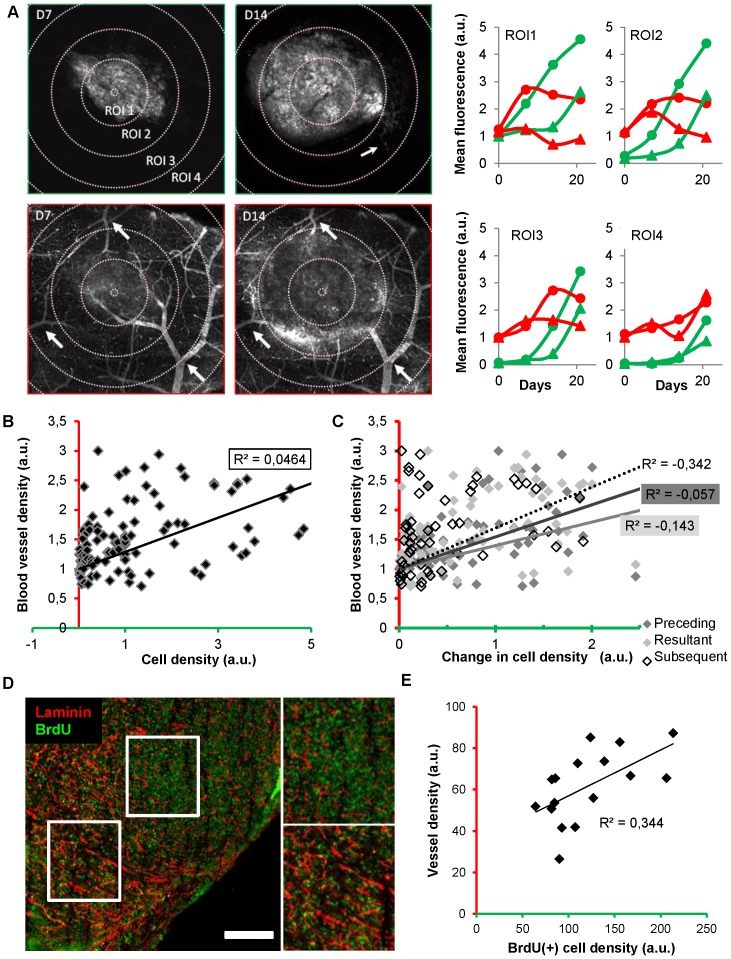
Quantitative analyses and lack of correlation between blood vessel density and tumor cell density. (**A**) Green (tumor cells) and red channel (vessel) images of the same plane showing the whole tumor section and its surrounding healthy brain obtained on D7 (day 7) and D14 respectively (depth, 40 µm below the brain surface). Small clusters of tumor cells were detectable (thin arrow in green channel, D14). Stable vascular landmarks (thick arrows in red channel) have been used to register the corresponding 3D image stacks. Initial tumor epicenter was systematically relocated to define 4 conserved circular regions of interests (ROI) (increment, 330 µm) whose mean fluorescence was monitored as an index of tumor cell density or blood supply. Evolutions of the cell density (green) and blood vessel density (red) in the 4 ROIs of two different mice (dots versus triangles). Note that local cell densities and local blood vessel densities are uncorrelated. (**B–C**) Correlative analysis of the data collected on mice (n = 7) over 4 sessions in 4 ROIs each time (**B**) Local blood vessel density is poorly correlated with local cell density (R^2^ = 0.0464). (**C**) The local changes in cell density ((Dfluo) over time (t2-t1 = Dt)) are also uncorrelated (R^2^<0) with the initial (t1) (black), resultant (t2) (grey) or subsequent blood vessel density (t3) (dashed line). (**D**) Immunostaining of vessels (anti-laminin, red) and BrdU positive cells (green) in a section of the whole tumor. White square boxes are enlarged on the right. (**E**) A poor correlation (R^2^<0.35) was found between vessel density and cell proliferation quantified from thresholded images of immunostained brain slices (n = 4) taken at D9 from 2 mice. Scale bars, 500 µm.

To search whether correlations could be found between vascular plasticity, vascular density, tumor cell density and proliferation we devise a technique to quantify our dynamic imaging data. To this end, we first established on GFP-U87 cell cultures that green fluorescence intensity was linearly correlated with cell numbers (R2>0.95, **Figure 3A**). Second, we confirmed that stable brain vascularization gave constant red fluorescence values over repeated imaging sessions (VC (Variation Coefficient) = 12% over 3 consecutive imaging sessions n = 19). Inter-animal variability was small (VC = 19%, n = 19, **Figure 3B**) including all sources of variations such as hardware stability, windows optical properties and animal repositioning or success of i.v. injection. We then used the mean fluorescence values on the green and red channels in concentric regions of interest (ROIs, **Figure 3D–E**) as indexes of local cell density and local blood vessel density, respectively. A circular field of 2.5 mm diameter was thus routinely analyzed over a depth of at least 200 microns. For each mouse, fluorescence was quantified for every individual ROI from the central (ROI1) to the peripheral (ROI4) relative to the tumor epicenter at 4 different times of development of the tumor (D0, D7, D14, D21). Despite implantation of calibrated spheroids of cells from the same culture passage, in the same cortical brain area of mouse littermates, tumor burden differed between animals on the first day of visualization (D0, VC = 58%, n = 19). For reliable comparisons, green fluorescence values recorded for each session were normalized for each animal to the one measured at the tumor core (ROI1) on the first day. Similarly, the red fluorescence inside the tumor was normalized to the mean fluorescence in surrounding healthy brain (ROI4). Relative changes of fluorescence values for equivalent ROIs were then averaged across animals. Exponential-like increase of tumor cell densities was observed in all regions with more or less delay at onset (**Figure 4A** **&** **5D**). Although the corresponding blood vessel densities increased asymptotically by 64±18% on average within 3 weeks, the dynamics of vascular changes were highly heterogeneous between ROIs and animals and appeared unrelated to the local changes in tumor cell densities (**Figure 4B** **,** **5C–D** **, S2, S3**).

**Figure 5:**
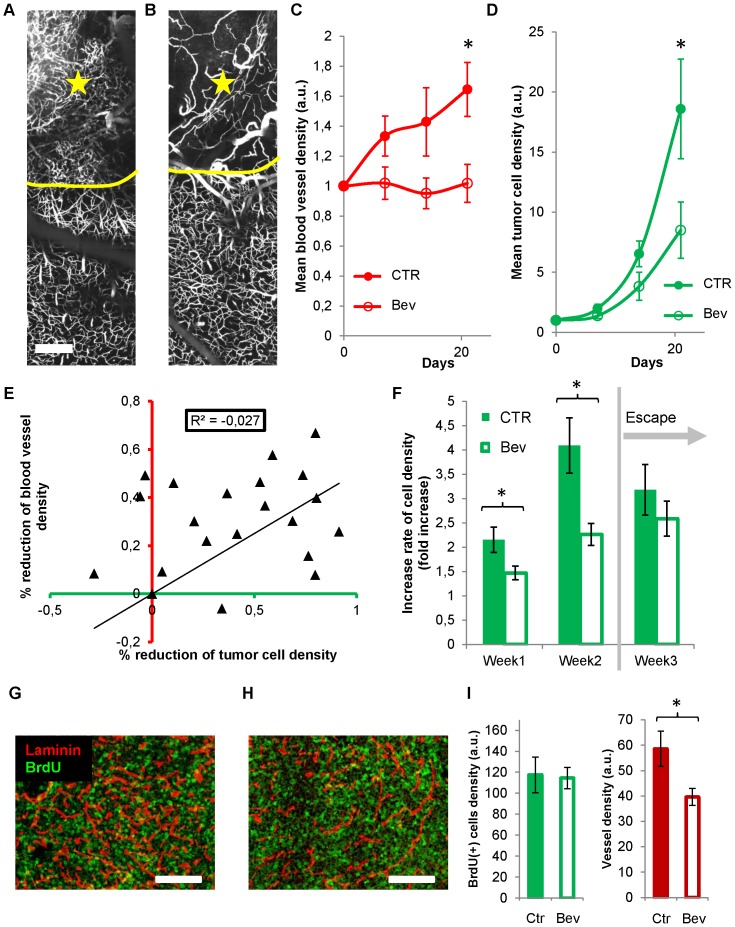
Dynamics of Bev treatment effects on tumor development and blood vessel density. (**A–B**) Example of 3D projections (over 200 µm) of high-resolution images of the microvascularization observed at the margin (yellow line) between tumor and healthy brain in an untreated mouse (**A**) and a mouse treated with Bev for 1 week (**B**). Tumor (star) exhibits a high density of tortuous vessels in the untreated compared to less dense and straighter vessels in the Bev-treated mouse. (**C–D**) Evolutions of the mean blood vessel density (**C**) and the mean tumor cell density (**D**) in corresponding brain volumes of control (n = 7) and Bev-treated (n = 7) mice (see methods). Tumor induced changes in blood vessel density are sustainably blocked by Bev (**C**) and mean tumor cell density is significantly reduced at day 21 (**D**) (*, p<0.05). (**E**) % reduction of blood vessel density relative to mean control values as a function of corresponding % reduction of tumor cell density. The absence of correlation (R^2^∼0) indicates that effect of Bev on tumor development is not mediated by its effect on blood vessel density. (**F**) Evolution of the increase rate of tumor cell densities in control and in mice treated with Bev for 3 weeks. Increase rate is expressed as the average of the ratios of fluorescence intensities recorded at one week intervals in the same areas. Increase rate is significantly reduced during the first 2 weeks but recovers the control increase rate on the third week despite a sustained blockade of vascular changes. (**G–I**) Immunostaining of vessels (anti-laminin, red) and BrdU positive cells (green) on D9 in untreated (**G**) or Bev-treated (**H**) tumors. Quantifications of BrdU(+) cells and blood vessels (**I**) in sections covering the whole tumor indicate that proliferation is not correlated with the amount of blood vessel density. Scale bars, 200 µm. Error bars represent SEM; (*, p<0.05).

### Weak correlation between tumor proliferation and local blood vessel density

We indeed found no strong correlation (R^2^ = 0.0464, p<0.0001) between local cell density and blood vessel density when considering the group of mice as a whole (**Figure 4B**). Analyzing data from individual subjects confirmed the overall independence of tumor growth with regard to blood vessel density since correlation was extremely weak in 5 out of 7 animals (−1.099<R^2^<0.298, p>0.05 n = 4 out of 5), and good only for the 2 others (R^2^ = 0.573 and 0.6718 respectively, p<0.0001 **Figure S2A**). We further investigated whether the strength of correlation was dependent on the level of tumor cell density (**Figure S2B**), on the stage of tumor evolution (**Figure S2C**), as well as on the location with respect to tumor center (**Figure S2D**). Linear correlation was poor in all cases, while no simple mathematical model performed better in describing the weak correlation between the two parameters (**Figure S2G**).

We also took into consideration the hypothesis that an increase in cell density might be either concomitant, precede or succeed to an increase in blood vessel density. We did not found correlations between changes in local blood vessel density either resultant ((R^2^ = −0.143, p<0.0001) preceding (R^2^ = −0.057, p<0.05) or subsequent (R^2^ = −0.342, p = 0.57) to changes in cell densities (**Figure 4C** **, Figure S2E, S2F**).

To finally search whether a correlation could be found between blood vessel density and proliferation we injected tumor bearing mice with BrdU two days prior sacrifice and performed immunolabeling experiments (**Figure 4D**). Since tumor heterogeneity can often bias data obtained from subject biopsies, we performed multiscale analysis of the bicolor fluorescence signals in the whole tumor area for sagital cryosections taken from the central part of the tumor. Similarly to *in vivo* results, vascular density was 1.7 times larger inside the tumor compared to surrounding healthy brain regions (57±7 a.u. compared to 34±5 a.u. n = 4), and a poor correlation (R^2^ = 0.344, p<0.05) was found between BrdU incorporation and the extent of local vascularization (**Figure 4E**) further supporting independence of tumor growth and vascularization. Independence of tumor progression with regard to blood vessel density does not appear to be a specific feature of U87 cells since it was also observed for the murine GL261 cell line grafted in C57Bl6 mice (**Figure S1**).

### Reduction in blood vessel density by Bev is without direct impact on tumor growth rate

**Figure 6:**
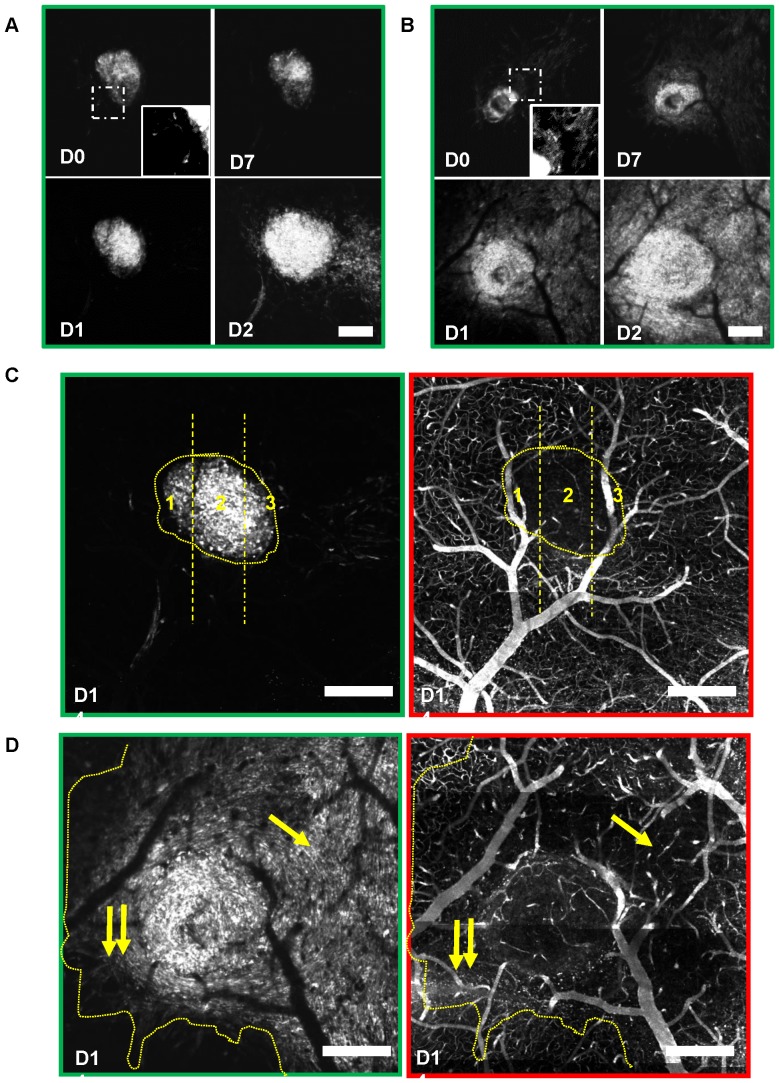
Heterogeneity of the Bev effect on tumor cell proliferation despite successful blockade of blood vessel formation. (**A–B**) Progression of the tumor under continuous Bev treatment. Mean projection images over a depth of 200 µm showing the evolution of tumor cell densities under Bev (10 mg/kg) treatment in 2 mice whose tumor burdens on D0 were similar (same mean fluorescence intensities within 6%, not shown). Treatment was initiated after the first visualization on D0. Tumor cells were more invasive and spread in B compared to A (see insets). Insets are the zoom of the region of interest outlined with dashed line. After 3 weeks the tumor cell density was more than 2 times larger in B than in A. (**C–D**) Independence of tumor progression from local blood vessel density. Max intensity projection of the tumor cell densities (green outline) at D14 in a 30 µm section of the tumors presented in A and B, side by side with the corresponding projection images of the vascular network (red outline). Tumor margins are highlighted with dotted yellow lines. Highest tumor cell densities are preferentially located in the central area (between vertical lines in C) with no bias toward the most vascularized sub-regions 1 or 3 compared to 2; High tumor cell densities are found in less vascularized subregions of the tumor (simple arrow versus double arrows in D). Scale bars, 400 µm.

To confirm the minor influence of blood vessel density on tumor progression in this model we tested whether angiogenesis could be blocked without modifying tumor growth rate. Mice (n = 13) were continuously treated with Bev for 3 weeks (10 mg/kg, i.v. every two days). One week treatment was sufficient to block vascular plasticity in tumor areas (3±2 vascular changes/mm3/24 h versus 71±19 in control tumors) and to massively reduce tumoral microvessel densities (**Figure 5A–B**). As a result, the average blood vessel density in tumor areas remained similar to the one observed at D0 for the whole duration of the treatment (**Figure 5C**). Under high dose of Bev [2], average tumor cell density was significantly reduced after 3 weeks (>50%, **Figure 5D**). As for untreated tumors, quantitative and correlative analysis of images revealed a complete lack of correlation between local blood vessel density and local cell density as well as between intersession changes in tumor cell density and the corresponding changes in blood vessel density (R^2^ = −0.0267, p = 0.28) (**Figure 5E**). By the end of the second week moreover, tumor cells escaped Bev treatment and recovered control growth rates despite a sustained blockade of angiogenesis (**Figure 5C–F**). BrdU immunostaining confirmed that tumor cell proliferation was similar in control and Bev treated animals despite a significant effect of Bev on tumor vascular density (**Figure 5G–I**). Interestingly, as illustrated (**Figure 6A–B**) the Bev tumor inhibitory effect was variable between animals and within given tumor areas cell densities were not related to the observed local blood vessel densities (**Figure 6C–D**). We finally found no indication of hypoxic necrotic area under Bev treatment, as no fluorescence holes were observed in tumor cells patterns regardless of tumor sensitivity to Bev (**Figure 6A–B**).

### Role of tumor micro­environment on GBM progression

The absence of correlation between cell densities and the levels of blood vessel density therefore suggests that the effect of Bev is either direct on tumor cells or indirect by inducing a non-permissive stromal environment. As VEGF is expressed both by brain cells [21] and by U87 cells [2], [22], first we monitored in real time the effect of exogenous VEGF and Bev on cultured U87 cell densities (**Figure S4A–B& S4D**) In culture, Bev in fact increased rather than decreased cell density by up to 12% in the first 48 h and still 7% in the following days. We thus concluded that a direct effect on tumor cells could not account for the 25% reduction in tumor growth observed *in vivo* in the same time line (**Figure S4B**). Cell tracking moreover showed that neither Bev, nor VEGF significantly affected U87 velocities, or migration on a given substrate (**Figure S4C**).

**Figure 7:**
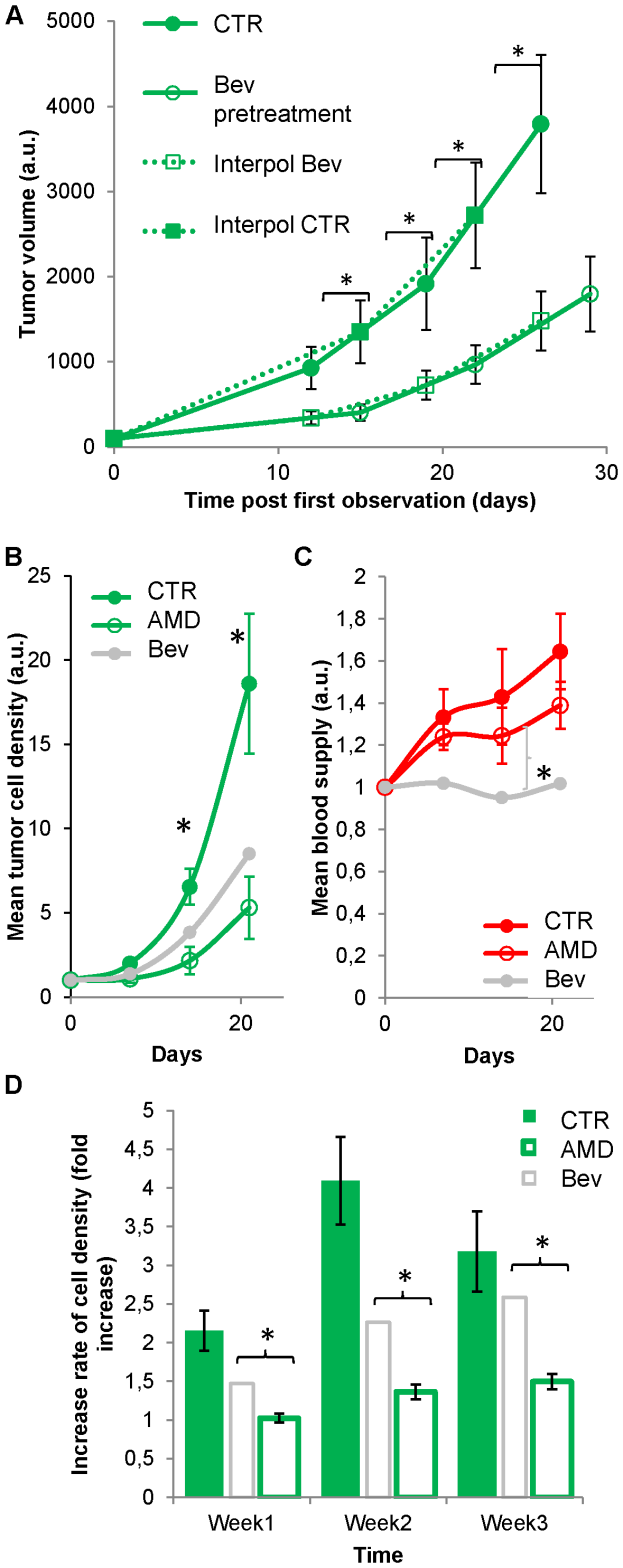
Contribution of the micro­environment on tumor development. (**A**) Evolution of the tumor volume in mice pretreated with i.v. injections every 2 days of PBS (n = 8) or Bev (n = 5) for 1 week before tumor implantation. Tumor volume was normalized on the first day of visualization (day 0) and interpolated linearly between imaging sessions to take into account day of visualization mismatches between the two groups of mice. Interpolated data are indicated with squares linked together with dashed lines. A significant reduction of tumor volume was observed on D14 until it reached 58% on D28 similar to what observed upon continuous Bev treatment. (**B–C**) Tumor development and blood vessel density under AMD3100 treatment to prevent signaling by Stromal Derived Factor 1 alpha. Evolutions of mean tumor cell density (**B**) and mean blood vessel density (**C**) in control (n = 7) or AMD3100-treated (n = 6) mice (*, p<0.05). The average evolution under Bev is reported in gray for comparison. Blood vessel density was not significantly affected by AMD3100 treatment and remained significantly larger than the one observed under Bev on D21. (**D**) Evolution of the average tumor increase rate in control conditions and under AMD3100 treatment. Ratios between fluorescence intensities recorded in the same areas at one week interval have been averaged and evolution of this average was followed over the 3 weeks of treatment. Bev data are reported in gray for comparison. Reduction of tumor size is almost twice larger under AMD3100 than under Bev.

Chemical signals from the environment play an important role in refractoriness or acquired resistance to anti-angiogenic therapy [23], [24]. We investigated whether Bev-induced depletion of stromal VEGF affected tumor progression. When GFP-U87 spheroids were grafted in mice pretreated with high doses of Bev for one week prior to tumor implantation, tumor growth was inhibited by 58±10% (n = 5 Bev, n = 8 Control) already at its earliest stage of development when angiogenesis is still low (**Figure 7A**).

**Figure 8:**
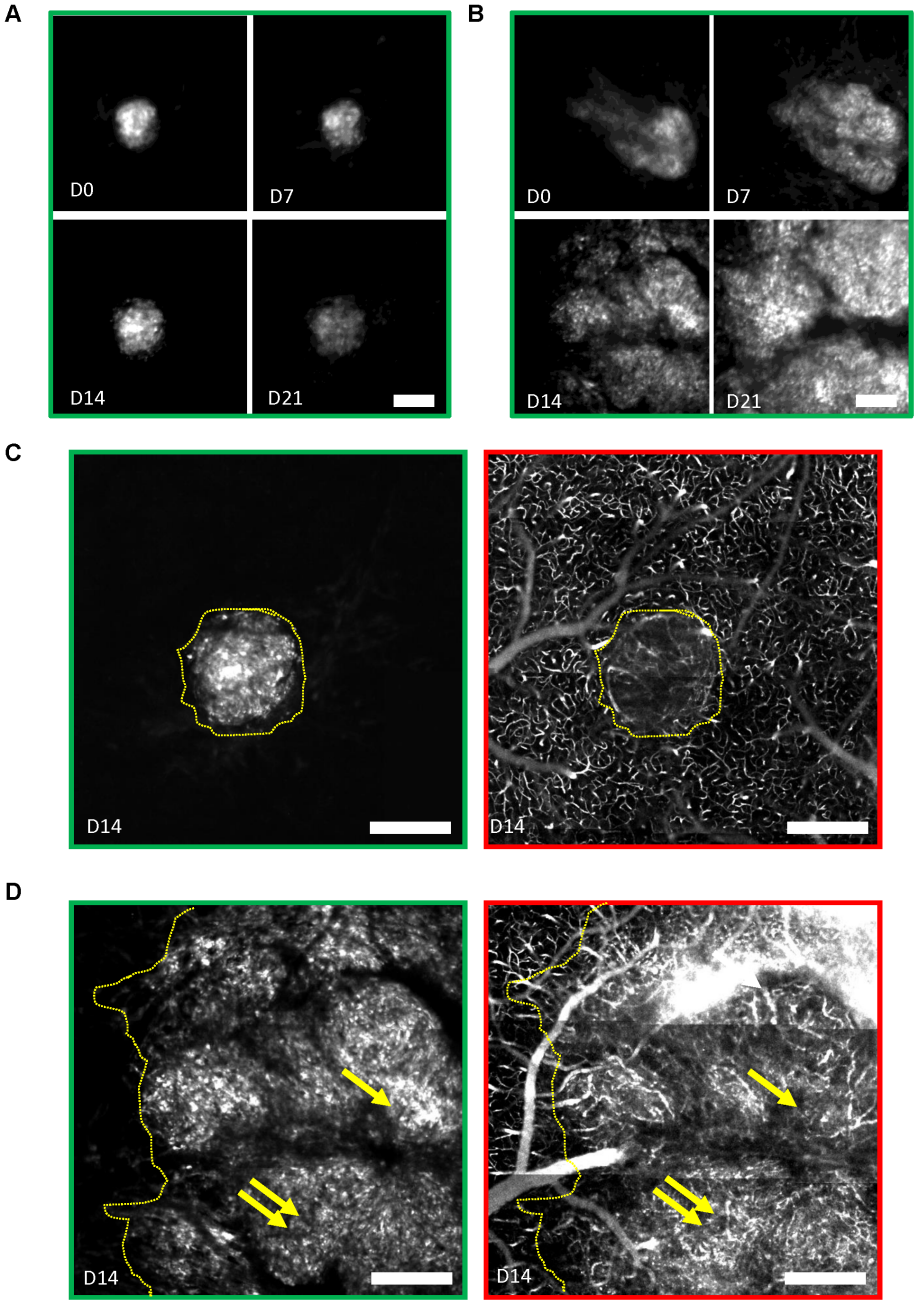
Sensitivity to AMD3100 is heterogeneous and independent of blood vessel density. (**A–B**) Mean projection images over a depth of 200 µm showing the evolution of tumor cell densities under AMD3100 (1.25 mg/kg twice daily) treatment in two mice whose tumor burden on D0 were similar (same mean fluorescence intensities within 16%, not shown). Treatment was initiated after the first visualization on D0. After 3 weeks, the tumor cell density was more than 2.5 times larger in B than in A. (**C–D**) Max intensity projection of the tumor cell densities (green outline) at D14 in a 30 µm section of the tumors presented in A, side by side with the corresponding projection images of the vascular network (red outline). Tumor margins are highlighted with yellow dotted lines. (**C**) Note the presence of tumor blood vessels compared to a similar size Bev treated tumor completely devoid of blood vessels shown in **Figure 6C**. (**D**) Dense tumor vascularization is even more obvious in the larger tumor. The highest vascular densities (double arrows) are not correlated with the highest tumor densities (single arrow). Scale bars, 400 µm.

As VEGF producing cells often release Stromal Derived Factor 1 alpha that modulates microenvironment and acts on tumor growth [18], [25] we used an antagonist of its CXCR4 receptor, AMD3100 (1.25 mg/kg, twice daily), as an alternative to Bev to reduce tumor growth. As observed with Bev, tumor sensitivity to AMD3100 was variable (**Figure 8**), but potent inhibition was observed on average (**Figure 7B**) without significant reduction in blood vessel density (**Figures 7C** **& S5**). Growth rate reduction was sustained and always 50% more pronounced than under Bev (**Figure 7B & 7D**). Unlike Bev, AMD3100 was equally efficient at the core and at the periphery of the tumor (**Figure S3**). For comparison with *in vivo* data we used linear regressions to extrapolate a possible cumulative effect of the drug during one week, but the modest direct effect observed in culture (**Figure S4E**) was unable to account for the 46% reduction observed *in vivo* after one week. This was in agreement with a major stromal action of AMD3100.

## Discussion

We have developed a dedicated orthotopic GBM model and an imaging methodology to study the progression of genetically labeled tumor cells with respect to the evolution of local micro-vascularization. The presented model was validated using xenografts of U87, a widely used human GBM cell line and GL261, a murine GBM cell line. The approach can be extended to virtually any cell line derived from patient biopsies previously transfected to stably express a fluorescent reporter.

The advantage of our model resides in the enforcement of 3D isotropic tumor growth orthotopically implanted in the superficial layers of the brain. Compared to previously described models in which the dura mater is removed [2], we here avoid unwanted deposit of sub- and supra-dural cells, whose proliferation is overwhelming in absence of 3D matricial constraints. As such, our model resembled patients' GBM, for whom extradural growth is rare [26]. This also likely explains the slower progression we observed compared to previously published models using the same cell line [2]. Moreover, injecting cells as spheroids helped to confine the GBM tumor within a determined brain area, since micro-regional heterogeneities inside the brain likely influence proliferation and invasion patterns [27], [28].

Efforts were made to improve the reproducibility of the imaging protocols and to take full advantage of quantitative fluorescence measurements. Whereas in most studies, tumor progression is estimated from the size of the long and short axis of the tumor [1], [2], [29], we have monitored the evolution of cell densities in ROIs that were conserved over the whole experiment. Besides providing an accurate evaluation of tumor progression, the methodology allowed the characterization of tumor heterogeneities as we systematically imaged a major part of the tumor volume. Whereas cells of the same passage were orthotopically implanted, we evidenced that the Bev tumor inhibitory effect was variable between animals and tumor areas, an effect unlikely due to heterogenous molecular profiles of the cells.

Our U87 GBM model revealed several lines of evidence indicating that increases in tumor cell densities were in fact not fueled by blood vessels as initially thought [4], [30]. First, in the absence of Bev treatment we found cases where tumor vascularization disappeared within a few days without affecting tumor cell densities. Second, although in some instances blood vessel density and tumor cell density showed some degree of interdependence as indicated by the low p value for Pearson correlation for two different GBM cell lines, we never observed a strong correlation between local cell densities and local blood vessel density as indicated by the weakness of their determination coefficients. Therefore an increase or reduction of blood vessel density cannot be predictive of a proportional change in cell density. Pharmacologically targeting blood vessel density might thus be a sub-efficient strategy to fight tumor. Supporting this view, tumor inhibition under Bev was not correlated with the amplitude of blood vessel density reduction. The maximal effect on tumor cells was observed in the first week of treatment at a time when angiogenesis was weak in the control group. Progression rate then recovered to control values within two weeks despite the sustained blockade of angiogenesis. This observation dismissed the hypothesis that tumor escape might result from activation of VEGF independent angiogenic pathways [9], [17], [31]. Although anti-angiogenic treatments are often reported to trigger tumor cell invasion [32], we did not find any evidence for increased invasiveness in our U87 model. However, cells that would have migrated far from the tumor cannot be detected due limitations of the imaging field of view; additional analysis based on tissue sectioning and vimentin immunochemical tumor cell detection should further document this issue. Finally under AMD3100, massive angiogenesis was maintained despite a strong reduction of tumor cell densities.

Our observations indicated that angiogenesis was not required to improve tumor metabolism in our experiments. No specific difference between central, intermediary or peripheral regions of the tumor was observed regarding the dependence of tumor growth on blood vessel density. This finding might be explained by the fact that GBM tumor cells grow inside the already densely vascularized healthy brain (**Video S1**) where 20% of the most oxygenated blood of the body is drained and distributed in real time according to the needs. Moreover, tumor cells have been shown to depend more on nutrients supply than on oxygen for their energy production [33]. GBM growth would therefore not require increased access to oxygen, but access to nutrients whose supply is already optimally organized in the brain to support energy expensive neuronal activity. A dense network of astrocytes [34] and a rich cerebrospinal fluid [35], [36] can provide nutrients in addition to blood. Angiogenesis independent tumor growth has already been reported [37], [38] and in that case average blood supply inside the tumor was even lower than in healthy brain.

Altogether our results obtained on a U87 tumor model support the challenging view that GBM growth is not directly related to blood vessel density. Yet the reason for such tumoral vascular remodeling remains to be elucidated. A possibility would be that tumor angiogenesis as well as tumor growth are promoted by inflammation [6], [39], [40], [41]. Inflammatory cells indeed shape the tumor micro-environment [42], [43] eventually promoting tumor growth [24], [40], [44], [45]. Our methodology should serve future studies to test whether the reported immuno-modulatory effect of anti-VEGF therapies [24], [40], [46], [47] or anti-SDF1 alpha therapies [45], [48] could account for the anti-angiogenic and anti-tumoral effects reported here.

## Supporting Information

Figure S1 **Lack of correlation between blood vessel density and tumor cell density in a syngenic orthotopic GBM model.** (**A**) Grafted GL261 DsRed tumor (red) visualized 16 days post-implantation. The vasculature is enlightened by the intravenous injection of Cascade-blue dextran 70 kDa (blue). Side images present a zoomed out version of each channel. (**B**) Absence of correlation between local tumor cell density and local intra-tumoral vascular density (R2<0. 1, n = 6 mice).
(TIFF)

Figure S2 **Weak correlation between tumor growth and blood vessel density in U87 GBM.** (**A**) Correlative analysis of the cell density and blood vessel density collected on a weekly basis for 3 weeks in all the different ROIs for each individual mouse (n = 7) according to the protocol described in **Figure 3**. Correlation is predominantly poor or inexistent (R^2^<0.3, 5 out 7 mice) which explained the average R^2^ = 0.046 observed in **Figure 4B** **.** (**B**) Correlation is poor irrespective of the range of tumor cell densities considered. Data presented in **Figure 4B** were binned using cell density ([0,1] dark gray; [1,2] light gray; [2,5] black); linear regression was determined in each bin. (**C**) Correlation is poor at every time point of tumor development. For each week, the dataset consisted of the measurements obtained on two consecutive sessions for all ROIs and all mice. (**D**) Correlation between cell density and blood vessel density as a function of distance to the center of the tumor. Correlation is not better in the most central area of the tumor (ROI1, black) compared to its periphery (ROI4, white). The gray value of data points is lighter from center to periphery. (**E**) Poor correlation between the local cell density and the resultant local blood vessel density (gray), between the local cell density and the local blood vessel density observed in the preceding session (black) as well as between the local cell density and the local blood vessel density observed in the subsequent session (white). Although weak, the correlation is better with the preceding local blood vessel density than with the resultant blood vessel density; it is worse with the subsequent than with the resultant blood vessel density. This suggests that blood vessel density does not increase to sustain the metabolic needs of tumor cells but that highly supplied areas trigger densification of tumor cells. (**F**) The amplitude of local changes of cell density measured between two consecutive time points are always positive and uncorrelated with the previous blood vessel density, uncorrelated with the resultant blood vessel density and uncorrelated with the subsequent blood vessel density. (**G**) Data presented in **Figure 4B** were not better fitted by other simple mathematical functions.
(TIFF)

Figure S3 **Regional efficacy of AMD3100 and Bev treatments on tumor cell density and tumor blood vessel density.** Data presented in **Fig. 5** **&** **7** for the whole tumor are here presented for every individual ROI from the central (ROI1) to the peripheral (ROI4) relative to the tumor epicenter. The largest differences between the two treatments on cell densities are observed in the most central ROIs whereas only Bev affects blood vessel density. Error bars represent S.E.M. and stars indicate significant differences relative to control values (p<0.05).
(TIFF)

Figure S4 **U87 cells behavior under VEGF, Bev and AMD3100 treatments.** (**A**) Comparative evolution of tumor cell densities in control and Bev (250 ng/ml) treated cultures (n = 2 independent experiments, 120 wells) (**B**) Ratios of mean cell densities in Bev versus control conditions (presented in A) are plotted over time. Green line represents the linear extrapolation of cell densities ratio until day 7. The modest increase in density observed *in vitro* cannot account for the 25% decrease in cell density observed *in vivo* after 1 week Bev treatment (gray arrow). (**C**) Instantaneous velocity (µm/min) of U87 cells measured every 30 min for 59 h under various conditions (control, VEGF or Bev). Note the absence of effect on cell migratory behavior (**D**) Average ratio of cell densities observed under treatment versus control conditions calculated over a 4.5 day experiment. Treatments were VEGF, Bev, a combination of Bev and VEGF (*: P<0,05; one-tailed Student t-test). (**E**) Evolution of the ratio between tumor cell densities observed in AMD3100 (10 µg/ml) treated cultures relative to control cultures. Linear extrapolation (green line) of the AMD3100 induced reduction in cell density indicate that the direct effect of AMD3100 on tumor cells (<20%) cannot account for the 46% reduction of tumor cell density observed *in vivo*.
(TIFF)

Figure S5 **Effect of AMD3100 and Bev on healthy and tumor blood vessel densities.** (**A,C,E**) Two weeks continuous treatment with either drug does not affect blood vessel density in healthy brain regions (AMD3100, A; Control = CTR, C; BEV, E). Max intensity projections of typical 30 µm thick sections of brain, before (D0) and 14 days (D14) after continuous treatment. (**B, D, F**) Similar projections as in (**A, C, E**) for tumor areas imaged 14 days after starting treatments. Tumor vascularization is strongly inhibited by Bev (**F**) and only weakly affected by AMD3100 (**B**) when compared to control tumor (**D**). Scale bar, 200 µm.
(TIFF)

Video S1 **3D volume rendering of a U87-GBM tumor.** Tumor cells are highlighted by GFP (green) and blood vessels by i.v. injection of Rhodamine Dextran 70 kD (red). Scale bar 400 µm.
(AVI)

Video S2 **Perivascular infiltration of U87-GBM cells.** Z-stack of horizontal images at 5 µm interval over 100 µm showing perivascular infiltration of the brain at the periphery of the primary tumor. Scale bar 100 µm.
(AVI)
