# Supplementary figures and images for: Dynamic Quantitative Intravital Imaging of Glioblastoma Progression Reveals a Lack of Correlation between Tumor Growth and Blood Vessel Density
Source: PLoS One. 2013 Sep 12;8(9):e72655. doi: 10.1371/journal.pone.0072655 (PMC3771993; doi:10.1371/journal.pone.0072655)

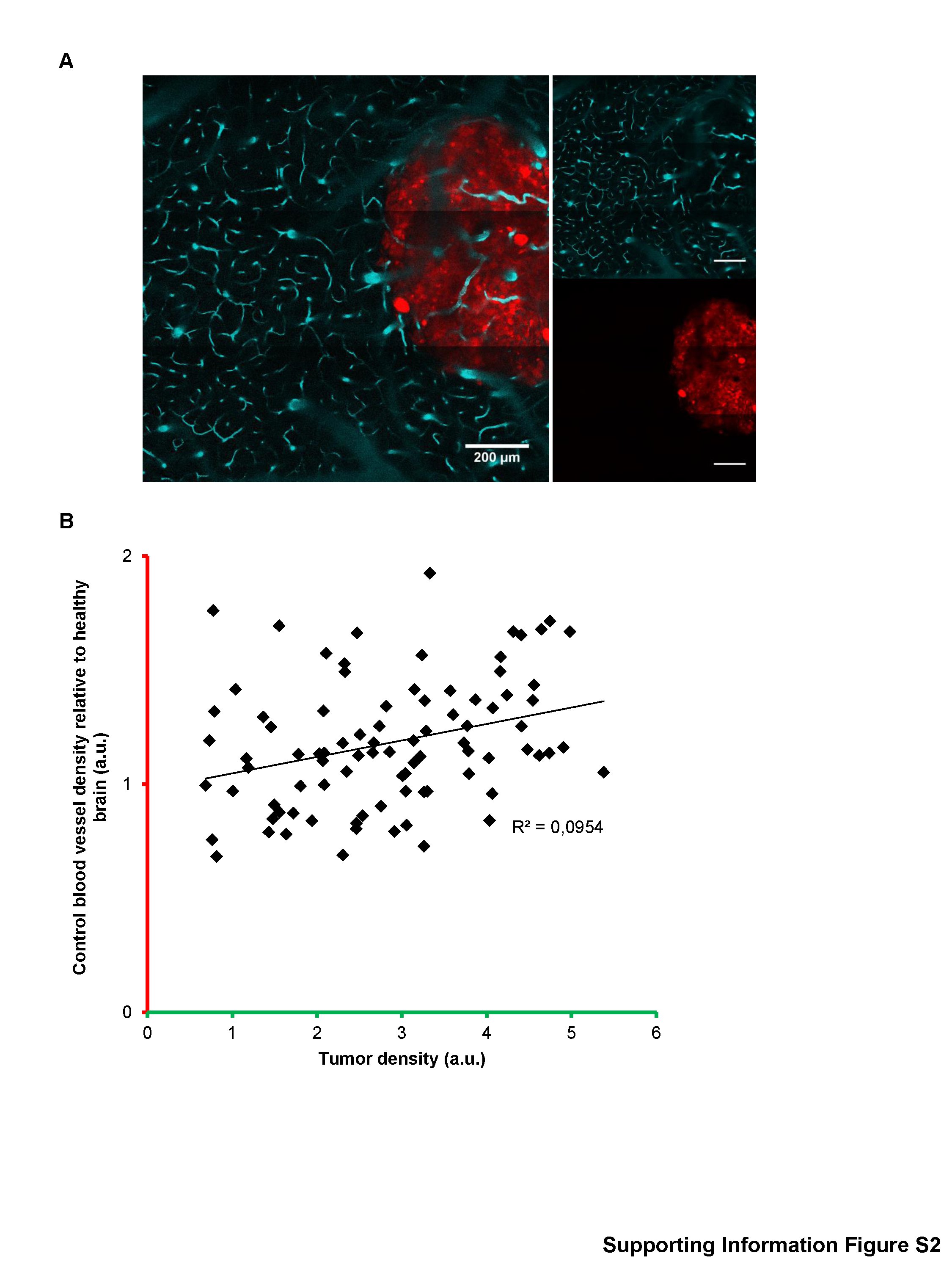

Supplement: Figure S1 — Lack of correlation between blood vessel density and tumor cell density in a syngenic orthotopic GBM model. (A) Grafted GL261 DsRed tumor (red) visualized 16 days post-implantation. The vasculature is enlightened by the intravenous injection of Cascade-blue dextran 70 kDa (blue). Side images present a zoomed out version of each channel. (B) Absence of correlation between local tumor cell density and local intra-tumoral vascular density (R2<0. 1, n = 6 mice). (TIFF) [file pone.0072655.s001.tiff]

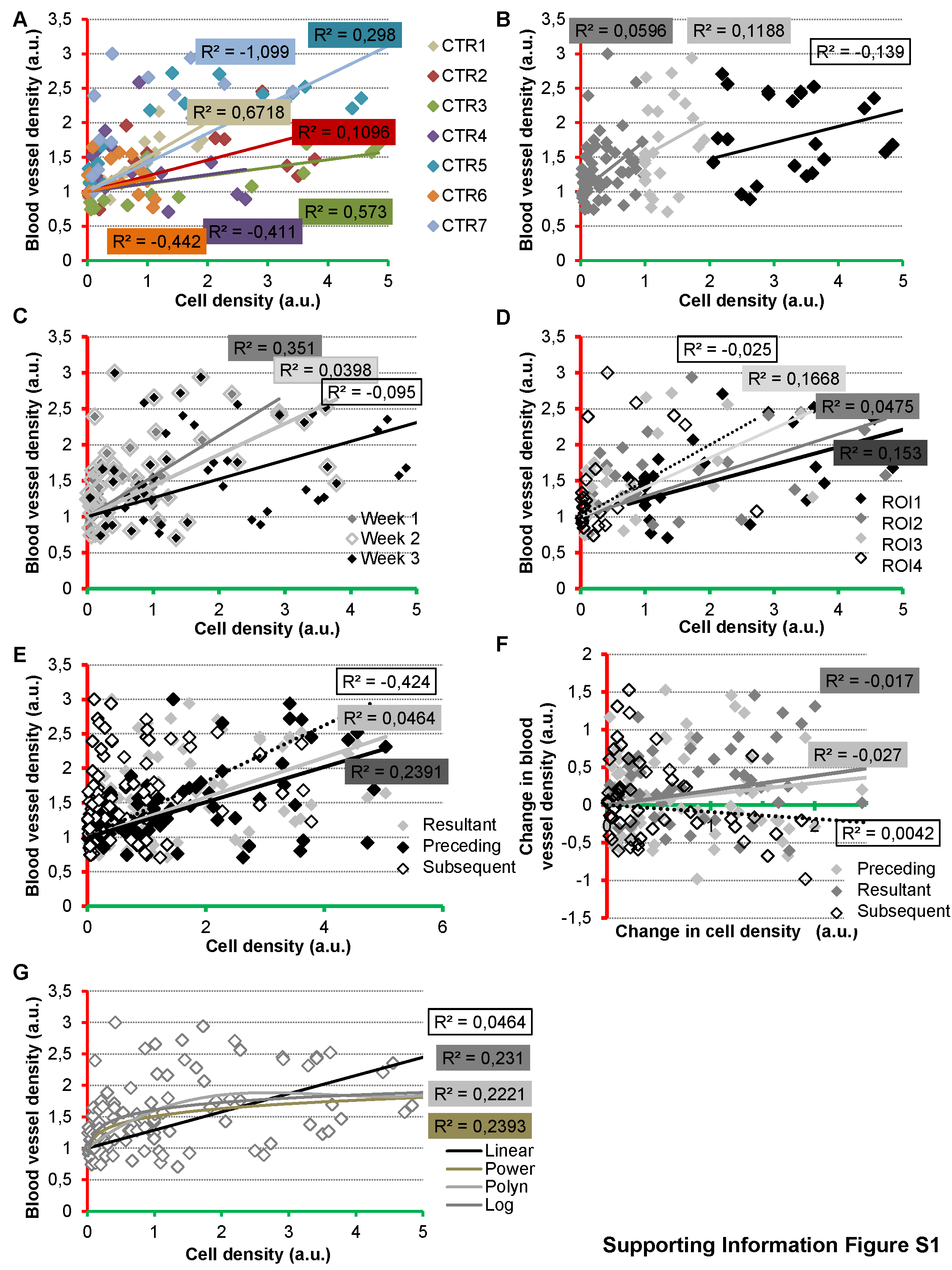

Supplement: Figure S2 — Weak correlation between tumor growth and blood vessel density in U87 GBM. (A) Correlative analysis of the cell density and blood vessel density collected on a weekly basis for 3 weeks in all the different ROIs for each individual mouse (n = 7) according to the protocol described in Figure 3 . Correlation is predominantly poor or inexistent (R2<0.3, 5 out 7 mice) which explained the average R2 = 0.046 observed in Figure 4B . (B) Correlation is poor irrespective of the range of tumor cell densities considered. Data presented in Figure 4B were binned using cell density ([0,1] dark gray; [1,2] light gray; [2,5] black); linear regression was determined in each bin. (C) Correlation is poor at every time point of tumor development. For each week, the dataset consisted of the measurements obtained on two consecutive sessions for all ROIs and all mice. (D) Correlation between cell density and blood vessel density as a function of distance to the center of the tumor. Correlation is not better in the most central area of the tumor (ROI1, black) compared to its periphery (ROI4, white). The gray value of data points is lighter from center to periphery. (E) Poor correlation between the local cell density and the resultant local blood vessel density (gray), between the local cell density and the local blood vessel density observed in the preceding session (black) as well as between the local cell density and the local blood vessel density observed in the subsequent session (white). Although weak, the correlation is better with the preceding local blood vessel density than with the resultant blood vessel density; it is worse with the subsequent than with the resultant blood vessel density. This suggests that blood vessel density does not increase to sustain the metabolic needs of tumor cells but that highly supplied areas trigger densification of tumor cells. (F) The amplitude of local changes of cell density measured between two consecutive time points are always positive and unc [file pone.0072655.s002.tiff]

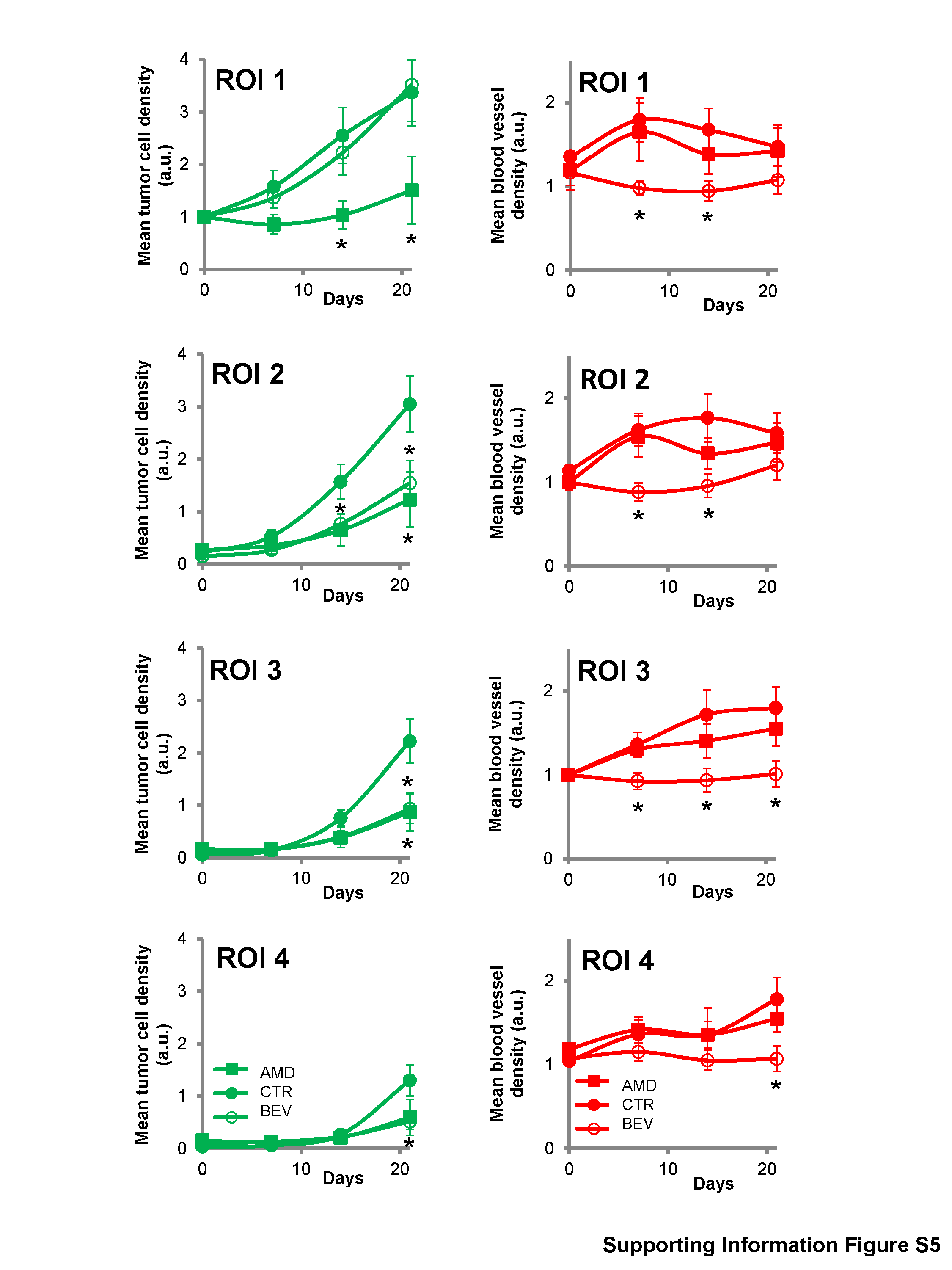

Supplement: Figure S3 — Regional efficacy of AMD3100 and Bev treatments on tumor cell density and tumor blood vessel density. Data presented in Fig. 5 & 7 for the whole tumor are here presented for every individual ROI from the central (ROI1) to the peripheral (ROI4) relative to the tumor epicenter. The largest differences between the two treatments on cell densities are observed in the most central ROIs whereas only Bev affects blood vessel density. Error bars represent S.E.M. and stars indicate significant differences relative to control values (p<0.05). (TIFF) [file pone.0072655.s003.tiff]

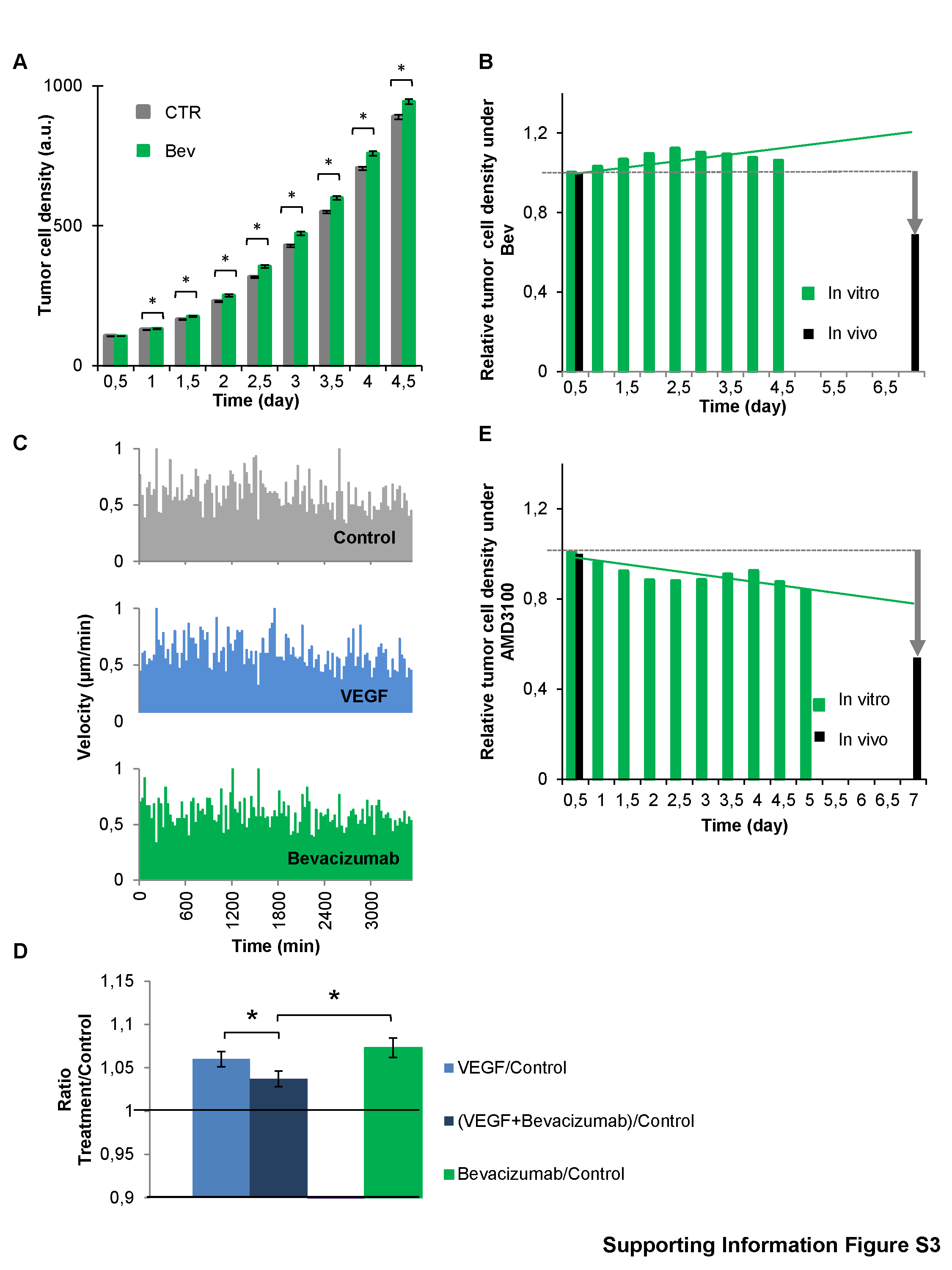

Supplement: Figure S4 — U87 cells behavior under VEGF, Bev and AMD3100 treatments. (A) Comparative evolution of tumor cell densities in control and Bev (250 ng/ml) treated cultures (n = 2 independent experiments, 120 wells) (B) Ratios of mean cell densities in Bev versus control conditions (presented in A) are plotted over time. Green line represents the linear extrapolation of cell densities ratio until day 7. The modest increase in density observed in vitro cannot account for the 25% decrease in cell density observed in vivo after 1 week Bev treatment (gray arrow). (C) Instantaneous velocity (µm/min) of U87 cells measured every 30 min for 59 h under various conditions (control, VEGF or Bev). Note the absence of effect on cell migratory behavior (D) Average ratio of cell densities observed under treatment versus control conditions calculated over a 4.5 day experiment. Treatments were VEGF, Bev, a combination of Bev and VEGF (*: P<0,05; one-tailed Student t-test). (E) Evolution of the ratio between tumor cell densities observed in AMD3100 (10 µg/ml) treated cultures relative to control cultures. Linear extrapolation (green line) of the AMD3100 induced reduction in cell density indicate that the direct effect of AMD3100 on tumor cells (<20%) cannot account for the 46% reduction of tumor cell density observed in vivo. (TIFF) [file pone.0072655.s004.tiff]

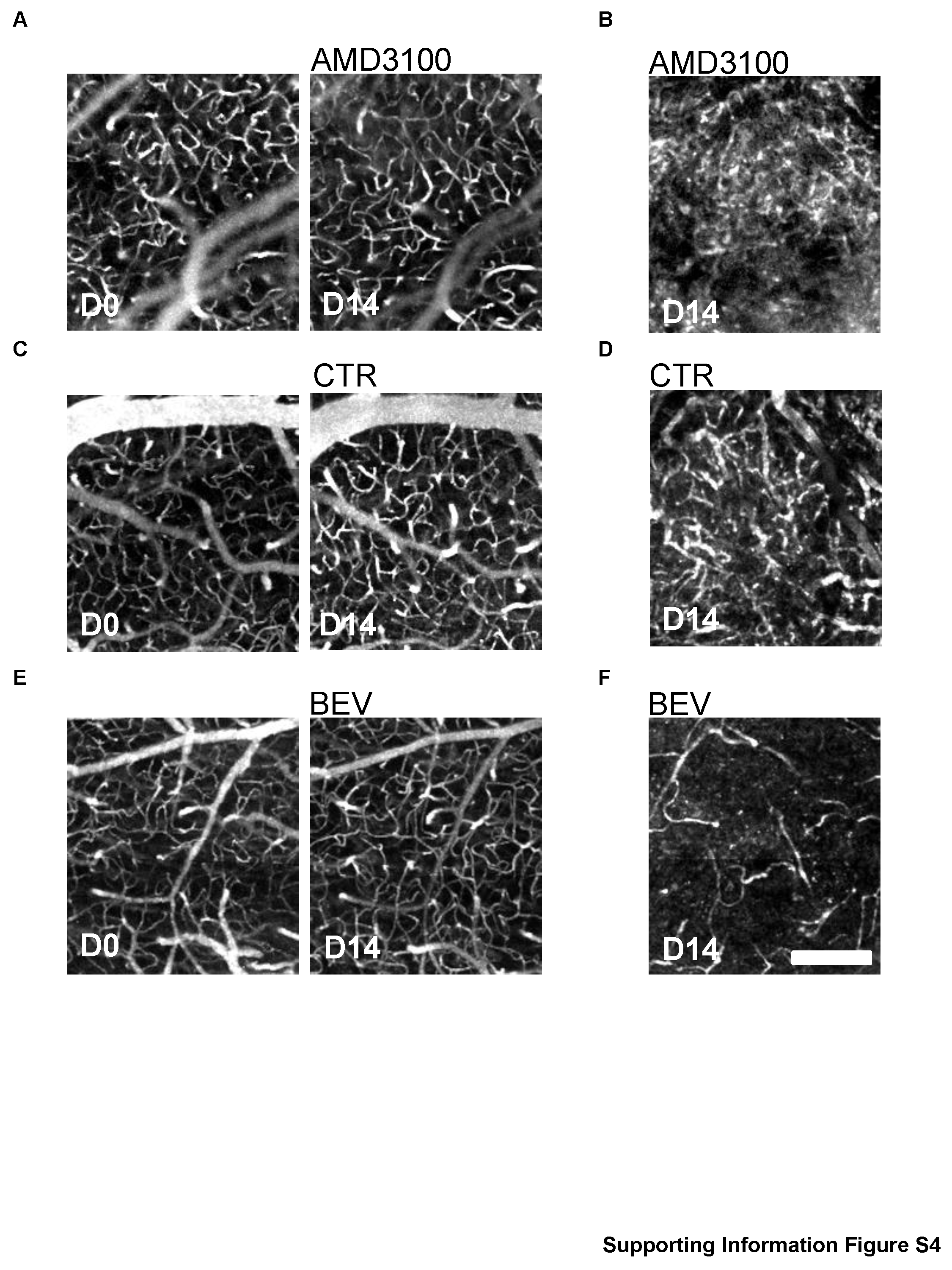

Supplement: Figure S5 — Effect of AMD3100 and Bev on healthy and tumor blood vessel densities. (A,C,E) Two weeks continuous treatment with either drug does not affect blood vessel density in healthy brain regions (AMD3100, A; Control = CTR, C; BEV, E). Max intensity projections of typical 30 µm thick sections of brain, before (D0) and 14 days (D14) after continuous treatment. (B, D, F) Similar projections as in (A, C, E) for tumor areas imaged 14 days after starting treatments. Tumor vascularization is strongly inhibited by Bev (F) and only weakly affected by AMD3100 (B) when compared to control tumor (D). Scale bar, 200 µm. (TIFF) [file pone.0072655.s005.tiff]
